# GPR139 agonist and antagonist differentially regulate retrieval and consolidation of fear memory in the zebrafish

**DOI:** 10.3389/fnins.2024.1461148

**Published:** 2024-12-05

**Authors:** Nisa Roy, Satoshi Ogawa, Sachiko Tsuda, Ishwar S. Parhar

**Affiliations:** ^1^Jeffrey Cheah School of Medicine & Health Sciences, Monash University Malaysia, Subang Jaya, Malaysia; ^2^Graduate School of Science and Engineering, Saitama University, Saitama, Japan

**Keywords:** GPR139, habenula, fear memory, calcium imaging, conditioned place avoidance

## Abstract

G protein-coupled receptor 139 (GPR139), a highly conserved orphan receptor, is predominantly expressed in the habenula of vertebrate species. Habenula is an ancient epithalamic structure, which is critical to comprehending adaptive behaviors in vertebrates. We have previously demonstrated the role of GPR139 agonists in fear-associated decision-making processes in zebrafish. However, how GPR139 signaling in the habenula modulates such adaptive behavioral responses remains unsolved. Fish centrally administered with a synthetic antagonist for human GPR139 (NCRW0005-F05) exhibited significant suppression of odorant cue (alarm substance, AS)-induced fear learning in the conditioned place avoidance paradigm. On the other hand, co-treatment with a GPR139 antagonist and a synthetic agonist for human GPR139 (JNJ-63533054) interrupted the fear conditioning process by significantly reducing locomotion during post-conditioning. Calcium imaging of acute brain slices showed a significant increase in peak amplitude of calcium transients in the habenula upon bath application of either a GPR139 antagonist or agonist. Furthermore, KCl-evoked calcium transients were reduced by the GPR139 antagonist and co-treatment of the GPR139 antagonist–agonist. These results suggest that the GPR139 antagonist did not block the inhibitory action of the GPR139 agonist in the decision-making process during the fear-retrieval phase; however, solitarily, it functions in governing the fear consolidation process via activation of the ventral habenula neurons in zebrafish.

## 1 Introduction

G protein-coupled receptors (GPCRs) are seven transmembrane receptors crucial for transducing signals from the extracellular environment into intracellular changes, which is critical to normal brain function (Wacker et al., 2017).^1^ GPR139 represents a brain-rich, structurally conserved orphan G protein-coupled receptor (Hu et al., 2009; Süsens et al., 2006). Although GPR139 exhibits relatively high binding affinities with several molecules such as the amino acids L-Trp, L-Phe, and adrenocorticotropic hormone (ACTH)/*α*-melanocyte-stimulating hormone (α-MSH)-related peptides (Nepomuceno et al., 2018; Liu et al., 2015), its endogenous ligand remains obscure. The role of GPR139 in the brain has been elucidated mainly in mice models with implications in neuropsychiatric diseases such as opioid addiction and schizophrenia (Wang et al., 2019; Dao et al., 2022; Reichard et al., 2021)_._ *Gpr139*-gene knockout (*Gpr139* ^−/−^) mice have recently provided evidence for the involvement of GPR139 signaling in the rewarding and analgesic effects of addictive drugs (Wang et al., 2019). GPR139 heterodimerizes with *μ*-opioid receptor to negatively regulate opioid receptor function (Wang et al., 2019). Another study using mouse models demonstrated the possible involvement of GPR139 signaling in the neuropsychiatric process with schizophrenia-like pathology (Dao et al., 2022). In mice lacking GPR139, loss of neuropsychiatric manifestations is driven by opioidergic and dopaminergic hyper-functionality (Dao et al., 2022). In addition, several synthetic selective agonists and antagonists have been developed to elucidate the role of GPR139 signaling (Dvorak et al., 2015; Shoblock et al., 2019). A study demonstrated the function of GPR139 agonists in dose-dependently protecting primary DA neurons against MPP^+^ toxicity (Bayer Andersen et al., 2016). *In vivo* approaches in mice warrant further validation primarily because of the multiple locations of GPR139-expressing sites in the brain. In humans and rodents, GPR139 is expressed in the habenula, lateral septal nucleus, basal ganglia, hypothalamus, and locus coeruleus (Süsens et al., 2006; Liu et al., 2015; Wang et al., 2019; Matsuo et al., 2005). While much of the work on GPR139 has been carried out in rodent models, its role in the teleost fish, zebrafish (*Danio rerio*), is beginning to be understood, particularly in relation to habenula biology. In zebrafish (*Danio rerio*), the orthologous gene of GPR139 (*gpr139*) is discretely expressed in the ventral part of the habenula (vHb) following fixing the adult brains in buffered 4% paraformaldehyde for 6 h (Roy et al., 2021; Pandey et al., 2018). Hence, the exclusive expression of *gpr139* in the habenula of zebrafish has inaugurated the fundamental role of GPR139 in habenula biology.

A bilateral pair of habenula nuclei display conserved neuroanatomical asymmetries associated with their functional specialization in particular cognitive processes (Bianco and Wilson, 2009). In mammals, the habenula comprises two subnuclei, the medial (MHb) and lateral habenula (LHb) (Aizawa et al., 2012; Concha et al., 2012). Similarly, in teleosts and amphibians, the habenula consists of the dorsal (dHb) and ventral (vHb), which correspond to mammalian MHb and LHb, respectively (Aizawa et al., 2005; Amo et al., 2010). In mammals, the habenula receives input from various parts of the limbic system and basal ganglia and communicates via the fiber tract, the fasciculus retroflexus. This enables it to send outputs to midbrain areas involved in the release of dopamine (the substantia nigra pars compacta and ventral tegmental area) and serotonin (the median and dorsal raphe nuclei) (Hikosaka, 2010). In primates and rodents, the unique position of the habenula and its influence on two monoaminergic nuclei aid it to participate in circuits that mediate highly integrative functions, such as reward response (Matsumoto and Hikosaka, 2007; Proulx et al., 2014), aversive responses (Hikosaka, 2010; Matsumoto and Hikosaka, 2007; Proulx et al., 2014; Matsumoto and Hikosaka, 2009), and associated cognitive processes such as decision-making (Hikosaka, 2010), learning, and memory (Wang et al., 2017; Song et al., 2017; Kobayashi et al., 2013). In zebrafish, dHb projecting to the interpeduncular nucleus (IPN) is responsible for controlling experience-dependent modification of aversive responses (Agetsuma et al., 2010), left–right-dependent decision-making, and social aggression in zebrafish (Cherng et al., 2020; Lee et al., 2010; Chou et al., 2016). Contrarily, vHb neurons process an aversive expectation value and are necessary for learning to avoid hazardous situations (Chou et al., 2016; Amo et al., 2014). In socially defeated zebrafish, *c-fos* expression is strongly induced in the vHb (Nakajo et al., 2020), suggesting that vHb is crucial for defining the aversive status of an individual. These results indicate that the habenula encodes an experience-dependent emotional decision-making process. We have previously shown a predominant expression of a neuropeptide, kisspeptin (Kiss1), in the vHb and its modulatory role in the odorant cue (alarm substance) induced fear-like responses in the zebrafish (Ogawa et al., 2014). We also demonstrated the possible involvement of Kiss1 in morphine-induced fear impairment (Sivalingam et al., 2020). Furthermore, the genetic ablation of *kiss1* impairs aversive learning in larval zebrafish (Lupton et al., 2017), confirming the role of the vHb neurons in the emotional decision-making process. However, the molecular mechanism of how vHb neurons accompany the learning process has not been fully elucidated.

We recently demonstrated the discrete expression of *gpr139* in the vHb and the possible role of GPR139 signaling in the contextual fear memory process in zebrafish (Roy et al., 2021). JNJ-63533054 is characterized as a potent agonist for zebrafish GPR139 receptors with an EC_50_ of 3.91 nM (Roy et al., 2021). Fish treated with a synthetic agonist for human GPR139 (JNJ-63533054) exhibited impairment of decision-making after fear conditioning in zebrafish (Roy et al., 2021). However, it remains unclear as to how the GPR139 signaling shapes the response patterns of vHb neurons during fear conditioning. In the present study, we first examined the effect of inhibition of GPR139 signaling on behavioral responses during fear conditioning. Second, the neural response profiles of vHb neurons during activation and inhibition of GPR139 signaling were examined by calcium imaging of acute brain slices. Our study is promising because it unveils the differential role of habenula orphan receptor GPR139 in multiple phases of fear conditioning.

## 2 Materials and methods

### 2.1 Animals and housing

Sexually mature (>6 months old) male, wild-type zebrafish (*Danio rerio*) were maintained in groups of 10 fish per 20 L freshwater aquaria (home tank) at 28 ± 0.5°C with a controlled natural photo regimen (14/10 h light/dark) at the Jeffrey Cheah School of Medicine and Health Sciences, Monash University Malaysia. The reason for using only male groups was to reduce variability, as female zebrafish tend to show higher anxiety behavior. The fish were fed with the Adult Zebrafish Diet (Zeigler, Gardners, PA, United States) twice daily. The developing embryos of zebrafish were obtained by placing several pairs of fish in a tank with glass marbles between 0900 and 1,000 h to allow mating. Fertilized eggs were siphoned from the tank and allowed to develop in culture dishes at 28.5 ± 1°C. After hatching, larvae were fed with ground Tetramin twice a day (Kitahashi et al., 2009).

### 2.2 Behavioral acclimatization

All experiments were carried out only after 1 week of fish acclimatization. The fish were anesthetized by immersion in water containing benzocaine (0.1 g benzocaine/200 mL water, Sigma) prior to injection and tissue dissection.

### 2.3 Dual-luciferase reporter assay

NCRW0005-F05, LP8, and JNJ-3792165 (Axon Medchem, Groningen, Netherlands) are commercially available antagonists, and Compound 1a and Takeda are commercially available agonists for human and other mammalian GPR139. To confirm whether NCRW0005-F05, LP8, and JNJ-3792165 act as antagonists to zebrafish GPR139, the binding of NCRW0005-F05, LP8, and JNJ-3792165 to the zebrafish GPR139 was examined by a dual-luciferase reporter gene assay in the presence of GPR139 agonist (JNJ-63533054) as described previously (see S1 Materials and Methods for details). To confirm whether Compound 1a and Takeda act as an agonist to zebrafish GPR139, the binding of Compound 1a and Takeda to the zebrafish GPR139 was examined by a dual-luciferase reporter gene assay (see S1 Materials and Methods for details).

### 2.4 Drug preparations, dilutions, and concentrations tested

Larval exposure: Seven dpf zebrafish larvae were treated with various concentrations (0.0625, 0.125, 0.25, 0.5, and 1% DMSO) of vehicle (control) or various concentrations (0.1, 0.2, 0.4, 0.8, and 1.72 mM in respective concentrations of DMSO) of NCRW0005-F05.

Adult injections: All experiments were carried out only after 1 week of fish acclimatization. The fish were anesthetized by immersion in water containing benzocaine (0.1 g benzocaine/200 mL water; Sigma) prior to injection and tissue dissection. The fish were injected intracranially with either 1 μL of human GPR139 antagonist (NCRW0005-F05) or 1 μL of 1% DMSO into the cranial cavity with a heat-pulled glass capillary micropipette attached to a microinjector (IM-9B; Narishige, Tokyo, Japan). The dose for the antagonist was chosen based on our toxicity assay in larvae. The final concentrations of DMSO contained in NCRW0005-F05 solution were minimized to 0.1 and 1% for 0.1 and 1 μg/g BW, respectively.

Fish were intraperitoneally injected with 1 μL of 0.1 μg/g GPR139 agonist (JNJ-63533054). The dose for JNJ-63533054 was chosen based on our previous *in vivo* study (Roy et al., 2021).

### 2.5 Effect of NCRW0005-F05 on locomotor activity in larvae

The effect of NCRW0005-F05 on locomotor activity was examined. Seven dpf zebrafish larvae were distributed individually to a 60-mm dish and treated with various concentrations of vehicle (control) or various concentrations of NCRW0005-F05. The larvae were kept under continuous light. The behavioral parameters tested to analyze free-swimming (hatched) larvae included swimming speed, swimming distance, and the location of the larvae in a Petri dish divided into inner and outer circles, allowing behaviors such as the thigmotactic response to be studied (Legradi et al., 2015). The thigmotactic response is the preference for being on the edge of a Petri dish over being in the center. The amount of time spent on the edge can be compared to the amount of time spent in the center. To achieve accurate tracking of the swimming behavior of individual larvae, SMART software (SMART V3.0, Pan Lab, Harvard Apparatus) was used to delineate the contour of the inner and outer zones with an equivalent spatial area within each well.

### 2.6 Intracranial administration of GPR139 antagonist (NCRW0005-F05)

Administration of the GPR139 antagonist (NCRW0005-F05) was carried out as previously described (Nathan et al., 2015; Ogawa et al., 2012). In brief, anesthetized fish were placed on a water-soaked sponge, and skulls were punctured with a 27 G X 1′ needle (Terumo, Shibuya-ku, Tokyo, Japan) at the midline of the telencephalon–diencephalon border. The fish were injected intracranially with either 1 μL of human GPR139 antagonist (NCRW0005-F05) or 1 μL of 1% DMSO into the cranial cavity with a heat-pulled glass capillary micropipette attached to a microinjector (IM-9B; Narishige, Tokyo, Japan). The dose for the antagonist was chosen based on our toxicity assay in larvae. The final concentrations of DMSO contained in NCRW0005-F05 solution are minimized to 0.1 and 1% for 0.1 and 1 μg/g body weight, respectively.

### 2.7 Intraperitoneal administration of GPR139 agonist (JNJ-63533054)

Administration of GPR139 agonist (JNJ-63533054) was carried out according to Samaee et al. (2017). Following anesthetization, fish were injected in the midline region between the pelvic fins using a Hamilton syringe (Hamilton, Reno, NV, United States) attached to a 30 G X ½ (0.3913 mm) needle (BD Precision GlideTM, Becton, Dickinson and Co., Franklin Lakes, NJ, United States), delivering 1 μL of 0.1 μg/g GPR139 agonist (JNJ-63533054). The dose for JNJ-63533054 was chosen based on our previous *in vivo* study (Roy et al., 2021).

### 2.8 Effect of the GPR139 antagonist (NCRW0005-F05) and co-treatment of NCRW0005-F05 and JNJ-63533054 on fear memory consolidation

**Figure 1:**
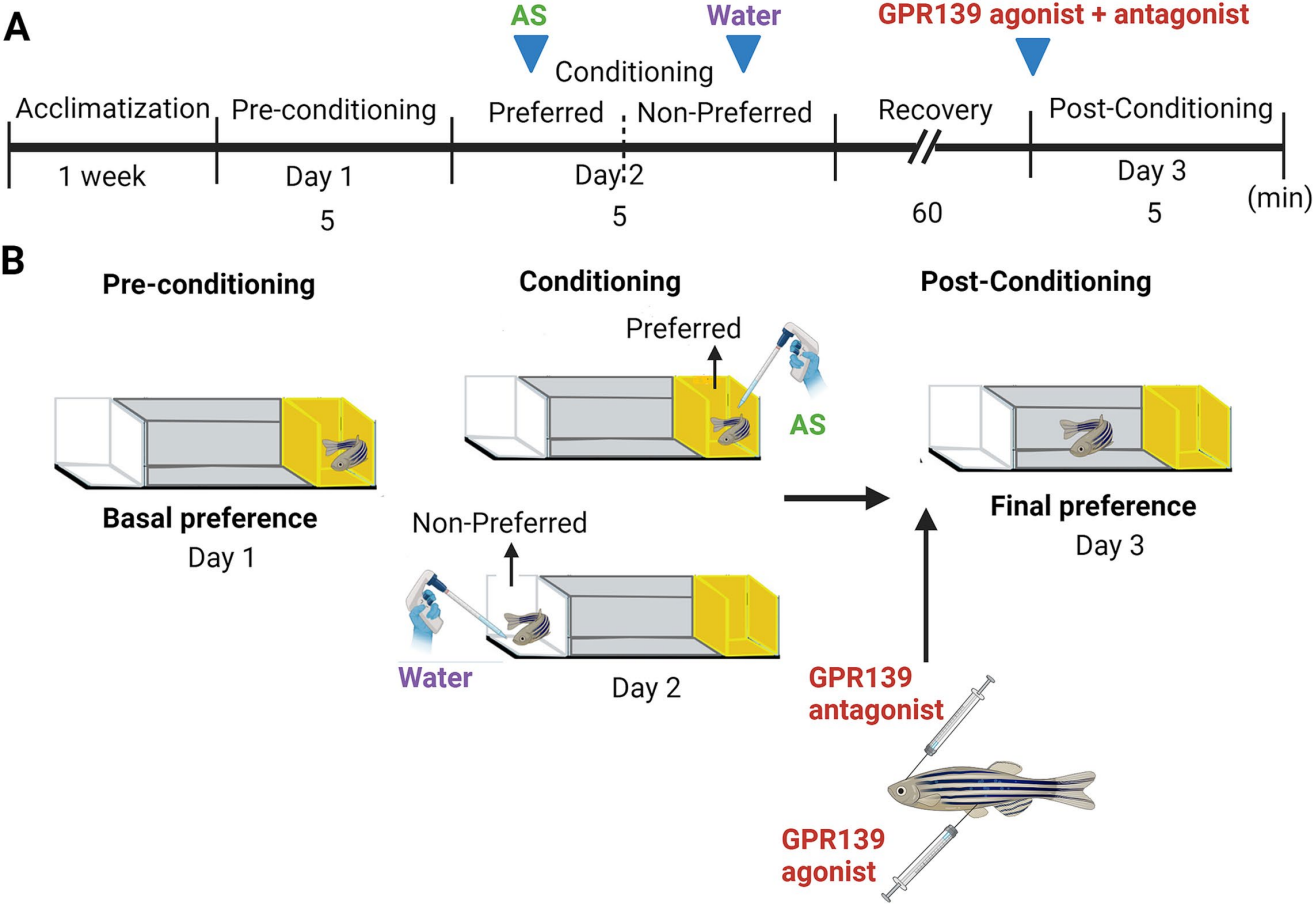
Alarm substance-induced fear conditioning and GPR139 antagonist and agonist treatment timeline during the conditioning. **(A)** Schematic of treatment timeline. After an acclimatization of 1 week, fish were given a choice of their preferred color (Day 1), either a yellow or white colored compartment (basal preference). On Day 2 (conditioning phase), fish were individually placed into the compartment, and after 5 min of acclimatization time, AS was delivered in water, followed by 5 min of video recording. The fish were then immediately transferred into the non-preferred compartment of the new experimental tank and exposed to 2 mL of distilled water (H_2_O) for 5 min. Intracranial injection of GPR139 antagonist and intraperitoneal injection of GPR139 agonist were then administered after 60 min of the recovery from the conditioned stimuli, and fish were transferred to their respective home tank. On Day 3 (post-conditioning phase), change in preference was assessed based on their total time spent in AS-paired (originally preferred) compartment as compared to the initial preference. **(B)** Schematic representation of conspecific alarm substance (AS)-induced fear conditioning paradigm. Adopted by Sivalingam et al. (2020) (Created with BioRender.com).

The effect of the GPR139 antagonist (NCRW0005-F05) and the co-treatment of NCRW0005-F05 and JNJ-63533054 on fear memory consolidation was assessed using an AS-induced conditioned place avoidance paradigm (Figure 1), previously established by Maximino and co-workers (Maximino et al., 2018) (see S2 Materials and Methods for details).

### 2.9 Calcium imaging

The fish brains were isolated, and 300 μm vibratome coronal sections were taken in HEPES-based Ringer’s solution (NaCl, 134 mM; MgCl_2_, 1.2 mM; CaCl_2_, 2.1 mM; KCl, 2.9 mM; HEPES, 10 mM; glucose, 10 mM) bubbled with 100% O_2_. These sections were incubated with 5 μM Oregon green 488 BAPTA-1 AM in Ringer’s solution supplemented with 0.1% pluronic F-127 at 25°C for 60 min, washed, and then incubated with Ringer’s solution for more than 30 min (Chou et al., 2016). For calcium recording, the brain sections were transferred into a glass-bottom dish equipped with perfusion tubes connected to a peristaltic pump. The Oregon green 488 BAPTA-1 fluorescence emitted from the tissues was detected and recorded by an Iris 9 Scientific CMOS (sCMOS) camera. Images were acquired by NIKON A1R with a 25×/0.95 NA water-immersion lens and 2×2 binned. An averaged intensity of the first 10s is considered F_0_. We then perfused an extracellular solution containing GPR139 agonists, antagonists, and KCl using a peristaltic pump. Following this, 1 min and 20 s after the application of the drugs, fluorescence changes from the first image were recorded for 60 s.

Basal pre-treatment fluorescence (F_0_) was recorded for 10 s after imaging had commenced using the NIS Elements (NIKON) software. For image analysis, ImageJ software is used. Peak fluorescence was selected from the highest fluorescent intensity readings at any time post-treatment. Change in fluorescent intensity (ΔF) was calculated by measuring the difference in intensity between current fluorescence and basal fluorescence (Chen and Huang, 2017). Data were analyzed using Student’s *t*-test and one-way analysis of variance (ANOVA). For counterstaining of the brain slices, the sections were fixed in buffered 4% paraformaldehyde at 4°C overnight. The sections were then washed and stained with 0.5 μg/mL of 4′,6-diamidino-2-phenylindole (DAPI) in PBS. Images of the sections were captured under an Axio V16 Fluorescent Microscope (Carl Zeiss).

## 3 Statistics

All behavioral data were analyzed using the Estimation Statistics Beta and the Statistical Package for the Social Sciences (SPSS, Version 24, IBM). All behavioral endpoints data were expressed as mean ± standard error of the mean (S.E.M.) and were compared using Student’s *t*-test, multi-two-group Cumming plot, shared control Cumming plot, and one-way and two-way ANOVA. Tukey’s multiple comparison test with a single pooled variance was reported with a 95% confidence interval level. For the dual-reporter luciferase assay, the results were analyzed using Prism (GraphPad Software, Inc., San Diego, United States) and are representative of three independent experiments conducted in duplicates. Luciferase responses were normalized as indicated, and the concentration–response curves were fitted using non-linear regression in a sigmoidal model with variable slope according to the standard procedure provided by GraphPad. Graphs were created using Estimation Statistics Beta and Prism.

## 4 Results

### 4.1 Pharmacological characterization of synthetic human GPR139 antagonists and agonists against zebrafish GPR139

The antagonistic activity of selective antagonists for human GPR139 (NCRW0005-F05, LP8, and JNJ-3792165) against zebrafish GPR139 was assayed using a dual-luciferase reporter assay with 30 nM of JNJ-63533054. The data showed that the dose–response curve for NCRW0005-F05 but not for LP8 and JNJ-3792165 effectively follows the shape of a receptor binding curve upon administration of a single concentration of GPR139 agonist (JNJ-63533054) and varying concentrations of NCRW0005-F05 with half-effective maximal concentration (IC_50_) values of 147.9 nM (Supplementary Figure S1). Analysis of responses of three replicates shows that NCRW0005-F05 induced luciferase activity with an inter-assay coefficient of variability of 14.12%. The binding affinity of selective agonists for human GPR139 (JNJ-63533054, Compound 1a, Takeda) on zebrafish GPR139 concludes that JNJ-63533054 but not Compound 1a and Takeda effectively binds and acts as reliable agonists to zebrafish GPR139 (Supplementary Figure S2). Analysis of responses of three replicates shows that JNJ-63533054 induced luciferase activity with an inter-assay coefficient of variability (CV) of 4.74% (Roy et al., 2021).

### 4.2 Behavioral effect of NCRW0005-F05

To validate whether NCRW0005-F05 exhibits any neurotoxicity, the effect of NCRW0005-F05 on the swimming pattern of larval zebrafish was examined. There were no significant differences in total distance (0.062% control, 0.1 mM treated, *p* = 0.952, Cohen’s *d* = 0.0341, *n* = 7; 0.125% control, 0.2 mM, *p* = 0.993, Cohen’s *d* = 0.0066, *n* = 7; 0.25% control, 0.4 mM treated, *p* = 0.324, Cohen’s *d* = 0.5312, *n* = 7; 0.5% control, 0.8 mM treated, *p* = 0.584, Cohen’s *d* = 0.0939, *n* = 7; 1% control, 1.72 mM treated, *p* = 0.122 Cohen’s *d* = 0.8302, *n* = 7) and total speed (0.062% control, 0.1 mM treated, *p* = 0.904, Cohen’s *d* = 0.0682, *n* = 7; 0.125% control, 0.2 mM, *p* = 0.303, Cohen’s *d* = 0.6461, *n* = 7; 0.25% control, 0.4 mM treated, *p* = 0.828, Cohen’s *d* = 0.1230, *n* = 7; 0.5% control, 0.8 mM treated, *p* = 0.570, Cohen’s *d* = 0.1524, *n* = 7; 1% control, 1.72 mM treated, *p* = 0.323 Cohen’s *d* = 0.4135, *n* = 7) between different doses of vehicle control and GPR139 antagonists (Supplementary Figures S3A,B). However, a significant difference in total time spent in the outer zone was observed among the two groups (0.125% control, 0.2 mM treated, *p* = 0.011, Cohen’s *d* = 1.4631, *n* = 7; 1% control, 1.72 mM treated, *p* = 0.0474, Cohen’s *d* = 1.078, *n* = 7) (Supplementary Figure S3C). In summary, these data indicate the presence of thigmotaxis restricted to some doses, without any general defects in other parameters in locomotion.

### 4.3 Effect of the GPR139 antagonist on fear learning in zebrafish

**Figure 2:**
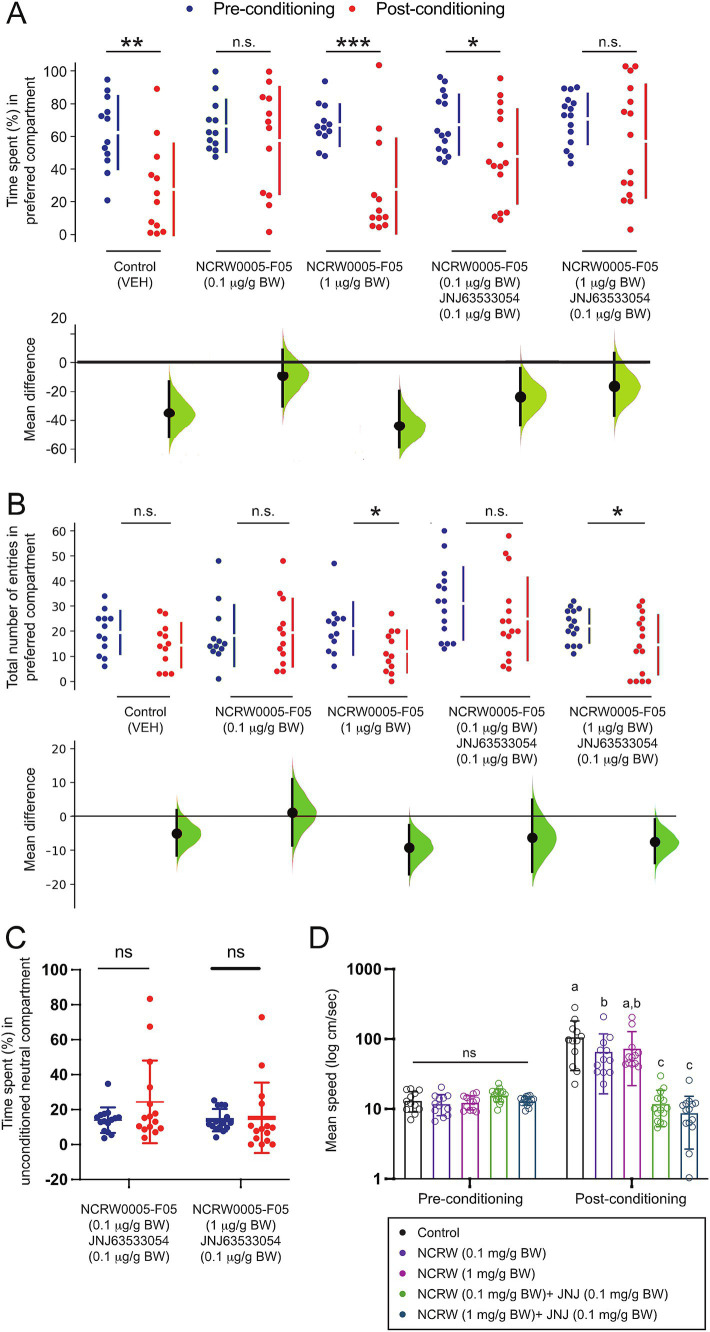
Effect of the GPR139 antagonist and co-treatment of the GPR139 antagonist and agonist on fear memory recall and avoidance. **(A)** During the post-conditioning (red dots), the time spent in the AS-paired compartment (originally preferred) was significantly reduced in vehicle control (1% DMSO, *p* = 0.0028, Cohen’s *d* = 1.1433, *n* = 12), fish treated with 1 μg/g BW of GPR139 antagonist (*p* = 0.0004, Cohen’s *d* = 1.4600, *n* = 12), and fish co-treated with 0.1 μg/g BW GPR139 antagonist and 0.1 μg/g BW agonist (*p* = 0.036, Cohen’s *d* = 0.7042, *n* = 15) as compared to pre-conditioning period (blue dots), indicating successful development of conditioned place avoidance, which was not seen in fish treated with 0.1 μg/g BW of GPR139 antagonist (*p* = 0.409, Cohen’s *d* = 0.2994, *n* = 12) and co-treated with 1 μg/g BW GPR139 antagonist and 0.1 μg/g BW GPR139 agonist (*p* = 0.173, Cohen’s *d* = 0.4485, *n* = 15). **(B)** There was a reduction in the number of entries to the conditioned compartment in fish treated with 1 μg/g BW of GPR139 antagonist (*p* = 0.0208, Cohen’s *d* = 0.8639, *n* = 12) and co-treated with 1 μg/g BW of antagonist and GPR139 agonist (*p* = 0.013, Cohen’s *d* = 0.740, *n* = 15) during the post-conditioning but not in the vehicle control (*p* = 0.152, Cohen’s *d* = 0.4790, *n* = 12), fish treated with 0.1 μg/g BW of GPR139 antagonist (*p* = 0.801, Cohen’s *d* = 0.1099, *n* = 12), and co-treated with GPR139 agonist and 0.1 μg/g BW of antagonist (*p* = 0.791, Cohen’s *d* = 0.403, *n* = 15). **(C)** During the pre- and post-conditioning phases, there was no significant difference in time spent within the neutral compartment in fish co-treated with 0.1 μg/g BW GPR139 antagonist and 0.1 μg/g BW GPR139 agonist and fish co-treated with 1 μg/g BW GPR139 antagonist and 0.1 μg/g BW GPR139 agonist. **(D)** Swimming speed was significantly reduced in both the co-treated groups (*p* < 0.0001) as compared to control or the fish treated with GPR139 antagonist alone. All behavioral data were analyzed using the Estimation Statistics Beta and the Statistical Package for the Social Sciences (SPSS, Version 24, IBM). All behavioral endpoint data were expressed as mean ± standard error of the mean (S.E.M.) and were compared using Student’s *t*-test, multi-two-group Cumming plot, one-way and two-way ANOVA, and Tukey’s test. *p < 0.05; **p < 0.01; ***p < 0.001; ns, not significant.

To elucidate the role of habenula GPR139 signaling in the modulation of fear conditioning, the effect of the GPR139 antagonists on conditioned place avoidance was examined. The time spent in the initially preferred compartment was significantly reduced after AS conditioning in the vehicle controls (*p* = 0.003, Cohen’s *d* = 1.1422, *n* = 12) and fish treated with 1 μg/g BW GPR139 antagonist (*p* = 0.0004, Cohen’s *d* = 1.4600, *n* = 12) (Figure 2A). On the other hand, in the fish treated with 0.1 μg/g BW of GPR139 antagonist, there was no difference in their time spent in the preferred compartment between pre- and post-conditioning (*p* = 0.409, Cohen’s *d* = 0.2994, Figure 2A, *n* = 12). The number of entries to the AS-paired compartment was significantly lower in 1 μg/g BW of GPR139 antagonist (*p* = 0.0208, Cohen’s *d* = 0.8639, *n* = 12) between pre- and post-conditioning (Figure 2B) but not in the vehicle control (*p* = 0.152, Cohen’s *d* = 0.4790, *n* = 12) and fish treated with 0.1 μg/g BW of GPR139 antagonist (*p* = 0.801, Cohen’s *d* = 0.1099, *n* = 12) (Figure 2B).

In fish co-treated with GPR139 agonist and 0.1 μg/g BW of the antagonist, the time spent in the preferred compartment was significantly reduced (*p* = 0.036, Cohen’s *d* = 0.7042, *n* = 15) after AS conditioning (Figure 2A). In contrast, in fish co-treated with 1 μg/g BW GPR139 antagonist and 0.1 μg/g BW GPR139 agonist, there was no difference in time spent in the preferred compartment between pre- and post-conditioning (*p* = 0.173, Cohen’s *d* = 0.4485, *n* = 15) (Figure 2A). Similarly, the number of entries to the AS-paired compartment was significantly lower in fish co-treated with 1 μg/g BW of antagonist and GPR139 agonist (*p* = 0.013, Cohen’s *d* = 0.740 *n* = 15) but not in fish co-treated with GPR139 agonist and 0.1 μg/g BW of antagonist (*p* = 0.791, Cohen’s *d* = 0.403, *n* = 15) after the fear conditioning (Figure 2B).

Given our previous observations that treatment with GPR139 agonists diminished the decision-making (increase in the time spent in the unconditioned neutral compartment) without interfering with fear memory consolidation (Roy et al., 2021), we next examined whether GPR139 antagonists could attenuate the effect of GPR139 agonists on conditioned place avoidance. In the co-treated groups, there was no significant difference in time spent within the neutral compartment between pre- and post-conditioning (Figure 2C). Interestingly, 0.1 ug/g BW of antagonist-treated fish reduced post-conditioned hyperactivity, but the co-treated group significantly reduced their swimming speed (cm/s) compared to the control during post-conditioning (*p* < 0.0001) (Figure 2D). Together, these results indicate that the GPR139 antagonists alone blocked conditioned place avoidance to the AS-paired compartment. However, the GPR139 antagonists failed to block the effect of GPR139 agonists on decision-making impairment.

### 4.4 Habenula neural responses to GPR139 antagonists and agonists

**Figure 3:**
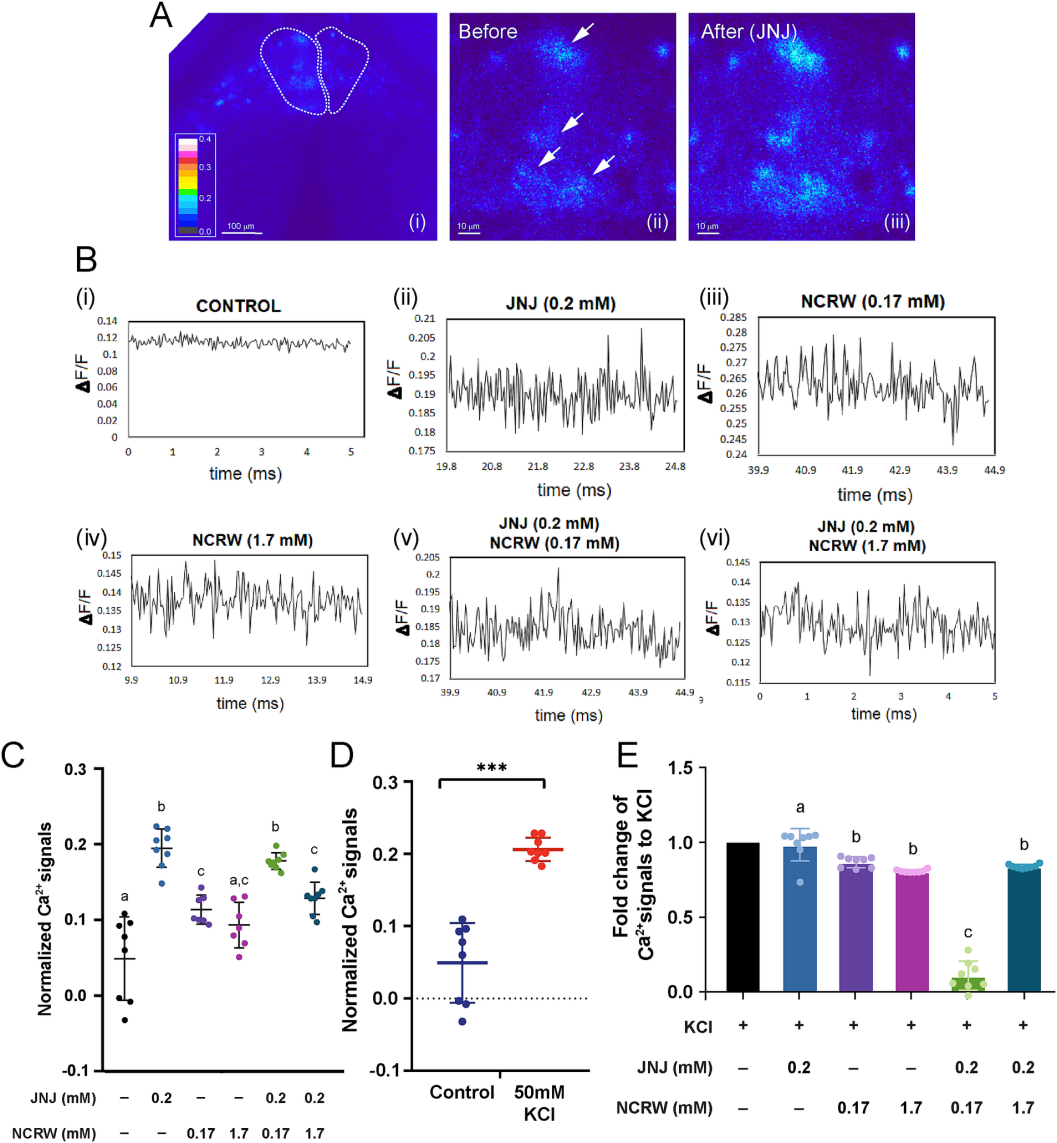
GPR139 regulates habenula neurons in acute brain slices. **(A)** Calcium imaging of habenula neurons in response to GPR139 compound. **(B)** Traces correspond to the cells upon (i) no treatment, (ii) 0.2 mM JNJ-63533054, (iii) 0.17 mM NCRW005-F05, (iv) 1.7 mM NCRW005-F05, (v) 0.2 mM JNJ-63533054 + 0.17 mM NCRW005-F05, and (vi) 0.2 mM JNJ-63533054 + 1.7 mM NCRW005-F05. **(C)** Average peak calcium signal in the habenula in presence of GPR139 agonist and antagonist treatment (control, average dF/*F* = 0.04892, *n* = 8; JNJ-63533054, average *dF/F* = 0.1948, *n* = 8, Cohen’s *d* = 1.6969; control, average *dF/F* = 0.04892, *n* = 8; 0.17 mM NCRW005-F05, average *dF/F* = 0.1135, *n* = 8, Cohen’s *d* = 1.2250; control, average *dF/F* = 0.04892, *n* = 8; 1.7 mM NCRW005-F05, average *dF/F* = 0.09326, *n* = 8, Cohen’s *d* = 0.9005; control, average *dF/F* = 0.04892, *n* = 8; 0.17 mM NCRW005-F05 + JNJ-63533054, average *dF/F* = 0.1778, *n* = 8, Cohen’s *d* = 1.9527; control, average *dF/F* = 0.04892, *n* = 8; 1.7 mM NCRW005-F05 + JNJ-63533054, average *dF/F* = 0.1288, *n* = 8, Cohen’s *d* = 1.3711). **(D)** Quantification of normalized calcium signal in habenula neurons shows a large increase in *dF/F* following bath application of 50 mM KCl (*p* < 0.0001, Cohen’s *d* = 2.2414). **(E)** Quantification indicates that there is a significant reduction in fold change in KCl-primed calcium transient by co-treatment with GPR139 agonist and 0.17 mM of GPR139 antagonist (*p* < 0.0001, R square = 0.9613). The spectrum for the pseudo-color representation in image **(A)** is used to represent calcium intensity. White arrows in images (**A** ii and iii) indicate habenula cells before and after GPR139 compound treatments. Scale bars: (**A** i), 100 μm; (**A** ii–iii), 10 μm. All reported results were expressed as mean ± standard error of the mean (S.E.M.) and were compared using unpaired Student’s *t*-test and one-way ANOVA. * *p* < 0.05; ** *p* < 0.01; *** *p* < 0.001; ns, not significant.

To elucidate the neuronal mechanism underlying the modulation of fear conditioning by GPR139 signaling in the habenula, we performed calcium imaging of acute brain slices after bath application of GPR139 antagonist and agonist (Figures 3Ai–iii,Bi–vi). The peak of calcium transients in the habenula neurons was significantly higher in the brain slices treated with 0.2 mM of GPR139 agonist (*p* < 0.0001, R square = 0.7676, *n* = 8) and 0.17 mM of GPR139 antagonist (*p* = 0.003, R square = 0.4002, *n* = 8) and co-treated with GPR139 agonist and GPR139 antagonist (0.17 mM and 1.7 mM; *p* < 0.0001, R square = 0.7288, *n* = 8) when compared to the control group, while there was no difference between 1.7 mM of GPR139 antagonist and control groups (*p* = 0.077, *n* = 8) (Figure 3C). The peak of calcium transients in the habenula neurons in slices treated with 0.17 mM and 1.7 mM GPR139 antagonist was significantly lower as compared to those treated with 0.2 mM GPR139 agonist (*p* < 0.0001, R square = 0.7848, *n* = 8) (Figure 3C). However, there was no difference between 0.17 mM and 1.7 mM of GPR139 antagonist groups (*p* = 0.813, *n* = 8). The peak of calcium transients in the slice co-treated with GPR139 agonist and 1.7 mM of GPR139 antagonist was significantly lower as compared to GPR139 agonist-treated group (*p* < 0.0001, R square = 0.6944, *n* = 8) while no such reduction was observed in the group co-treated with GPR139 agonist and 0.17 mM of GPR139 antagonist (*p* = 0.873; Figure 3C, *n* = 8). On the other hand, calcium transients in the co-treated group (GPR139 agonist and 0.17 mM GPR139 antagonist) were higher than the group treated with 0.17 mM of GPR139 antagonist alone (*p* = 0.003, R square = 0.8379, *n* = 8). However, there was no such difference between the 1.7 mM GPR139 antagonist and the co-treated group (*p* = 0.926, *n* = 8) (Figure 3C).

To further identify the impact of GPR139 signaling on calcium dynamics of the habenular neurons, we observed the effect of the GPR139 antagonist and agonist on calcium transients in the habenula cells upon activation by 50 mM KCl application. The calcium transients in the habenula cells treated with 50 mM KCl were significantly (*p* < 0.0001, Cohen’s *d* = 2.2414, *n* = 8) higher than those in the slices without KCl treatment, thus prompting depolarization of habenula neurons by the KCl application (Figure 3D). The KCl-primed calcium transients were significantly reduced by co-treatment with GPR139 agonist and 0.17 mM of GPR139 antagonist (*p* < 0.0001, R square = 0.9613) (Figure 3E).

## 5 Discussion

We demonstrate that fish co-treated with GPR139 antagonist and agonist did not reverse GPR139 agonist-induced impairment of decision-making during the fear-retrieval phase; however, surprisingly, in fish treated with the GPR139 antagonist, fear conditioning, particularly fear consolidation, was disrupted. In brain slices, GPR139 agonist-induced and KCl-primed calcium transient in the habenula was suppressed by the GPR139 antagonist, suggesting that the GPR139 antagonist regulates fear consolidation via modulation of habenula neural activities in the zebrafish.

Our previous study showed that the zebrafish treated with the GPR139 agonist, JNJ-63533054, exhibited avoidance of fear-conditioned and unconditioned compartments (Roy et al., 2021), suggesting that activation of GPR139 signaling in the habenula may have compromised the conditioning or decision-making process. However, it remains unclear as to how GPR139 signaling in the vHb neurons could modulate the decision-making process. In addition, the effect of synthetic GPR139 antagonists on behavioral phenotypes has not been elucidated in any animal species, mainly due to a lack of characterization of GPR139 antagonist candidate with the blood–brain barrier (BBB) permeability that is suitable for *in vivo* assay (Vedel et al., 2020). We first screened whether the three commercially available human GPR139 antagonists, namely, NCRW0005-F05, LP8, and JNJ-3792165, exhibit antagonistic activity to zebrafish GPR139 against 30 nM of GPR139 agonist (JNJ-63533054). The luciferase assay revealed that the antagonists did not exhibit any pharmacologically antagonistic effect on zebrafish GPR139. In larval zebrafish, there was no major effect of NCRW0005-F05 on locomotor activities at lower doses, while the treatment with NCRW0005-F05 at a higher dose induced a minor thigmotactic effect, indicating anxiety-like behavior. We have previously established a protocol for intracranial administration of drugs in adult zebrafish (Ogawa et al., 2012). In addition, zebrafish has been proposed as a promising *in vivo* model for assessing the delivery of natural products, fluorescence dyes, and drugs across the BBB (Jeong et al., 2008). For example, upon administration of Sulfo-NHS-biotin (443 Da) into the heart of adult zebrafish, massive leakage of biotin was observed in the median habenula (Jeong et al., 2008). Hence, we then tested the effect of NCRW0005-F05 on conditioned place avoidance. Our previous study showed that administration with GPR139 agonist does not affect fear acquisition or consolidation, but it diminished avoidance of the conditioned (both AS- and non-AS-paired) compartments in zebrafish (Roy et al., 2021). This indicates that the GPR139 agonist could mainly interrupt the retrieval phase of the fear conditioning. In the present study, the avoidance of the AS-paired compartment was diminished when the fish were treated with the lower dose of GPR139 antagonist, indicating that the fear consolidation phase but not the retrieval phase could be interrupted. In mammals, LHb inactivation abolishes reactive defensive response (freezing/avoidance) when threat and safety memory compete during retrieval (Velazquez-Hernandez and Sotres-Bayon, 2021; Sachella et al., 2022). On the other hand, optogenetic inhibition of the LHb in male rats during cue and unconditioned stimulus did not affect freezing to the context (Sachella et al., 2022), suggesting that inhibition of the LHb during the entire training is required to impair the contextual component of fear conditioning (Velazquez-Hernandez and Sotres-Bayon, 2021). Multiple pieces of evidence demonstrated the role of habenula in fear conditioning; for instance, endocannabinoid control of medial habenula to interpeduncular neurons exerts a necessary role in the expression of aversive memories (Soria-Gomez et al., 2015). In addition, excitatory transmission to the lateral habenula is critical for encoding and retrieval of spatial memory (Mathis et al., 2015). Similarly, in zebrafish, inactivation of the vHb is sufficient to impair active avoidance learning (Amo et al., 2014). Hence, GPR139 signaling could play differential roles in multiple phases of fear conditioning. In mice, neither context nor cued memories could be independently expressed when conditioning takes place without the proper activity of the LHb (Sachella et al., 2022). However, memory expression is evident when both contextual and cued components of fear conditioning are reactivated by the presentation of the cue in the conditioning context (Sachella et al., 2022). Hence, if the activity of the LHb is disrupted during fear conditioning and learning, memory retrieval requires the synergy of contextual and cue information (Sachella et al., 2022). In our study, treatment with GPR139 agonist/antagonist was given before the fear-retrieval stage; hence, it can be hypothesized that training (cued fear acquisition) and consolidation (contextual memory development) had taken place normally. A synergy of cue and contextual information is processed via activation of habenula to process the conflict; however, how this process could have been disrupted by activation or inhibition of GPR139 signaling remains to be further elucidated.

In zebrafish, administration of GPR139 agonist disrupts the contextual component of fear conditioning but not freezing and fear learning (Roy et al., 2021). Although we have not examined the neural activity of the habenula during fear conditioning in zebrafish, the basal levels of vHb neural activity might have already been high during the fear conditioning process, similar to LHb in mammals (Sachella et al., 2022). Hence, it can be speculated that the GPR139 agonist could have induced hyperactivation or inactivation of the vHb neurons. On the other hand, exposure to the GPR139 antagonist also induced a minor activation of the habenula neurons, while there was no effect on the fear memory retrieval phase. This suggests that the GPR139 antagonist-induced stimulation of the habenula could be insufficient to induce hyperactivation of the habenula. Conceptually, these observations suggest that the fear conditioning seems to be taking place adequately, but the habenula might have failed to retrieve AS-paired context as an aversive memory when the GPR139 antagonist was administered. On the other hand, in the GPR139 agonist-treated group, the habenula might have failed to synergize the cue (AS and water) and contextual (yellow and white) information because of possible hyperactivation of vHb by GPR139 signaling activation. Nevertheless, this is still hypothetical and remains to be further validated in examining habenula neural activity during fear conditioning.

We then assessed how NCRW0005-F05 regulates habenula neural physiology. In the present study, habenula neurons exhibited increased peak intensities of calcium transients in response to activation of the GPR139 receptor by agonist treatment, which might be associated with its inhibitory action on fear conditioning (Roy et al., 2021). While co-treatment with the GPR139 antagonist did not reverse the GPR139 agonist-induced impairment of decision-making, it suppressed the fear consolidation phase. These contradictions suggest that the GPR139 agonist and antagonist could independently act on the GPR139 signaling pathway. Then, 0.17 mM but not 1.7 mM of the GPR139 antagonist treatment suppressed GPR139 agonist-induced and KCl-primed calcium transients in the habenula, indicating that the action of GPR139 agonist on the habenula neural activities is partially blocked by GPR139 antagonist. GPR139 is a dual-specificity receptor capable of binding to Gi/o and Gq/11 classes upon application of 10 μM JNJ-63533054 in GPR139-transfected HEK293 cells (Stoveken et al., 2020). However, GPR139 primarily engages the Gq/11 but not the Gi/o pathway to activate adenylyl cyclase and inhibit the G protein inward rectifying potassium (GIRK) (Stoveken et al., 2020). The relevance of GPR139-mediated G_q/11_ signaling to counteracting MOR in the endogenous setting was accomplished by electrophysiological recordings from medial habenular neurons, where MOR and GPR139 are co-expressed (Stoveken et al., 2020). Application of the MOR agonist DAMGO significantly dampened neuronal firing. However, pre-treatment with GPR139 agonist completely blocked DAMGO’s effects on firing, suggesting GPR139 signaling via G_q/11_ is necessary and sufficient for counteracting MOR-mediated inhibition of neuronal firing (Stoveken et al., 2020). Given the similar signaling mechanisms of MOR and dopamine D2 receptor (D2R), recent evidence suggests that in addition to MOR, GPR139 also inhibits the dopamine D2 receptor (D2R) actions *in vitro* (Dao et al., 2022), which further impact behavioral manifestations by enhancing dopaminergic signaling in mice lacking GPR139 (Dao et al., 2022). Antagonist affinities can also vary depending on the agonist they are counteracting and the presence or absence of allosteric ligands. This could also be partly derived from the competitiveness of the GPR139 agonist and antagonist due to their difference in affinities against GPR139 and downstream signaling cascades (Wacker et al., 2017; Weis and Kobilka, 2018). In fact, several antagonists against GPCR exhibit different affinities for a particular receptor that couple to form complexes with more than one G protein (Baker and Hill, 2007). In mammals, GPR139 is modulated by amino acids L-Phe and L-Trp and several endogenously expressing neuropeptides (Nøhr et al., 2017). We envision that if there is an ongoing, basal, or tonic level of response due to the actions of potential endogenous GPR139 ligands, a competitive antagonist such as NCRW0005-F05 could reduce the response of the agonist by competing for a binding site on the receptor. Alternatively, the failure to block the effect of the GPR139 agonist by GPR139 antagonist on decision-making could be due to different routes of their administration (different action modes or pharmacokinetics). Interestingly, there was a significant alteration in locomotor activity (speed but not total distance swam) when the fish were co-treated with GPR139 agonist and antagonist. This effect was only found when the fish were co-treated with GPR139 agonist and antagonist, as the treatment with GPR139 agonist alone failed to suppress locomotor activity in our previous study (Roy et al., 2021), although the mechanism underlying the suppression of locomotor activity remains unclear. This suggests that co-treatment may have interrupted not only locomotion but also the fear-conditioning process itself. In addition, control fish exhibited hyperactivity during post-conditioning, which may be due to higher sensitivity to AS-induced aversion and, in turn, might have resulted in escalated locomotion. In mice, GPR139 and dopamine D2 receptor (D2R) are colocalized in several brain regions, including the LHb, lateral septum, interpeduncular nucleus, and medial raphe nuclei (Wang et al., 2019). In HEK293 cells co-expressing D2R and GPR139, the calcium response from the co-expressed receptors could be antagonized by either a D2R or GPR139 antagonist (Wang et al., 2019). Furthermore, in *Gpr139*^−/−^ mice, administration of the D2R antagonist completely suppressed the locomotor hyperactivity, suggesting the locomotor activity by GPR139 is modulated via dopaminergic signaling (Dao et al., 2022). In addition, neurons in the LHb project directly or indirectly to dopaminergic neurons in mammals (Hong et al., 2011; Omelchenko et al., 2009). In zebrafish, although the expression of dopamine receptors in the vHb remains unclear (Roy et al., 2021; Pandey et al., 2018), we have previously demonstrated the possible connections between the vHb-median raphe (MR) and dopaminergic neurons (Abdul Satar et al., 2020). Hence, co-administration of the GPR139 agonist and antagonist might have affected the downstream of the habenula pathway via other neurotransmitter systems, which remains to be further elucidated.

It is interesting to consider the observed phenotypes in GPR139 agonist and antagonist-treated fish from the perspective of neuronal circuitry. GPR139 is exclusively expressed in the ventral part of the habenula (vHb). The habenula is involved in reward-based decision-making. Animals with habenula lesions become hyperactive and distractible and make motor responses prematurely in a reaction-time task (Lee and Huang, 1988). LHb responds to the negative value of a stimulus that contributes to the suppression of body movements, leading to an aversive outcome, which is evidenced in rats with habenula lesions showing impairments in avoidance learning (Jhou et al., 2009). Stress-induced activation of LHb neurons (Wirtshafter et al., 1994) in rats has been shown to inhibit dopamine neurons, which subsequently leads to the suppression of motor activity (Seligman, 1972). Many of these observations lead us to speculate that the behavioral deficits we observe are related to the deregulation of habenular function. The behavioral anomalies seen upon loss of GPR139 in mice, including hyperactivity and PPI deficits, are reminiscent of schizophrenia symptoms in humans (Dao et al., 2022). Genetic variations in the *GPR139* locus have been linked to symptoms of inattention in attention-deficit hyperactivity disorder and schizophrenia (Castellani et al., 2014). Conceptually, these neurological disorders associated with cognitive deficits are also implicated with decision-making impairment.

In summary, our study showed that the central administration of a synthetic GPR139 antagonist diminished fear conditioning. However, it could not block the inhibitory action of the GPR139 agonist on fear memory retrieval (decision-making) when they were co-administered, indicating that NCRW0005-F05 could be considered as a partial antagonist. This finding implies that the GPR139 antagonist suppresses the fear consolidation phase, and the GPR139 agonist and antagonist could independently act on the GPR139 signaling pathway. Calcium imaging on acute slice culture showed that the GPR139 agonist and antagonist increased the amplitude of calcium transients in the habenula neurons, while the effect of the GPR139 agonist on calcium transients was only partially reduced by the GPR139 antagonist. It could imply that the GPR139 agonist induced hyperactivation or inactivation of the vHb neurons, while the GPR139 antagonist also induced a minor activation of the habenula neurons. In addition, fish that were co-administered with the GPR139 agonist and antagonist exhibited reduced locomotor activity. Taken together, these results suggest that GPR139 signaling in the habenula plays a differential role in multiple phases of fear conditioning via modulation of neural activities of habenula neurons during fear learning in zebrafish.

## Data Availability

The original contributions presented in the study are included in the article/Supplementary material, further inquiries can be directed to the corresponding authors.

## Footnotes

^1^Habenula orphan G-protein coupled receptors in the pathophysiology of fear and anxiety https://www.sciencedirect.com/science/article/pii/S0149763421005017?via%3Dihub
