# Supplementary material for: GPR139 agonist and antagonist differentially regulate retrieval and consolidation of fear memory in the zebrafish
Source: Front Neurosci. 2024 Dec 5;18:1461148. doi: 10.3389/fnins.2024.1461148 (PMC11665214; doi:10.3389/fnins.2024.1461148)
Supplement: Supplementary file 1 [file Data_Sheet_1.pdf]

Supporting Information for
GPR139 agonist and antagonist differentially regulate retrieval and consolidation of fear memory in the zebrafish

Nisa Roy[1,2], Satoshi Ogawa[2*], Sachiko Tsuda[3], Ishwar S. Parhar[4*]
[1]National Institutes of Health (NIH), United States
[2] Monash University Malaysia, Malaysia
[3] Saitama University, Japan
[4] University of Toyama, Japan

*** Correspondence:**
Ishwar S. Parhar and Satoshi Ogawa
Jeffrey Cheah School of Medicine and Health Sciences,
Monash University Malaysia,
47500 Bandar Sunway,
Selangor, Malaysia.
Tel: +603-5514-6306; Fax: +603-5514-6341
E-mail: ishwarparhar@gmail.com, ishwar@ctg.u-toyama.ac.jp
satoshi.ogawa@monash.edu

This PDF file includes:

Supporting text
Methods S1 to S2
Figures S1 to S3
SI References

Supporting Information Text

S1 Materials and Methods

Dual-luciferase reporter assay.

A pcDNA3.1(+) expression vector containing an open reading frame of zebrafish gpr139 cDNA (pc_zfGPR139, Clone ID, ODa58136D) was obtained from GenScript Ltd (Hong Kong). HEK293T cells were maintained in Dulbecco's modified Eagle's medium (DMEM; GIBCO, Auckland, NZ) supplemented with 10% fetal bovine serum (FBS), 0.1×penicillin–streptomycin solution (iDNA, Kuala Lumpur, Malaysia) under 5% CO2. One day before transfection, cells were plated in 24-well plates in the media without penicillin-streptomycin. Co-transfection of pc_zfGPR139 or pc_hGPR139 (100 ng/well) with pSRE-Luc (100 ng/well; Stratagene, La Jolla, CA), and pRL-TK vectors (25 ng/well; Promega, Madison, WI) was carried out with Lipofectamine 2000 transfection reagent (Invitrogen; Termo Fisher Scientifc, Inc.) overnight according to the manufacturer's instructions. The cells were serum-starved in the media with 0.5% FBS for 18–20 h and then treated with the control (30 nM JNJ-63533054) or various concentrations of the NCRW0005-F05, LP8, and JNJ-3792165 in 0.5% DMSO followed by 30 nM human GPR139 agonist (JNJ-63533054) in the media for 6 h. The cells were harvested and lysed with passive lysis buffer, then analyzed immediately using a 96-well plate luminometer (Infinite M200pro, Tecan, Switzerland). Luciferase activity and Renilla luciferase in the cell extracts was determined using the Dual-Luciferase Reporter Assay System (Promega) according to the manufacturer's instruction. The value of luciferase for each lysate was normalized to the Renilla luciferase activity. The relative transcriptional activity was converted to fold induction above the corresponding control value (n-fold).

S2 Materials and Methods

AS-paired conditioned place avoidance paradigm.

Alarm substance is a potential stimulus for avoidance conditioning that sensitises anxiety-like behavior after a single exposure and elicits behaviors, such as erratic movements and freezing (1). The effect of GPR139 agonist and antagonist on conditioned place avoidance was examined using the AS-induced fear response model. AS solution was prepared according to the protocol implemented previously (2). Briefly, male fish were anesthetised by submerging them in ice-cold water, and fifteen shallow cuts were made on the right trunk of the zebrafish with a razor blade, and the cuts were washed with 5 mL ice-cold distilled water. This was then repeated on the left trunk of the fish to obtain a total of 10 mL AS solution per fish.

Experimental apparatus. A test tank (31 cm L×16 cm W×20 cm H) is divided into three chambers using a lightweight board made of corrugated plastic with yellow or white (13 cm L×16 cm W×20 cm H) and a grey central partition (31 cm L×5 cm W×20 cm H) and two sliding guillotine-type doors (16 cm×20 cm) (1). The partition allows the water to move throughout the tank and the AS solution can be diffused throughout the tank in a minute after application. Another tank (31 cm L×16 cm W×20 cm H) used as an acclimatizing tank, was similarly divided as above but by a transparent divider to allow conspecific to visualize in order to minimize isolation stress. The top view of fish behavior was recorded by a video camera (positioned approximately 1 m above the tank), between 1100 and 1600 h with a similar temperature (28±0.5 °C) and lighting (802.4 lx illumination) condition to the home tank. Captured video data were analyzed using automated tracking software, SMART (3).

Acclimatization. A week prior to the behavioral study, fish were randomly taken from the home tank and transferred to a regular fish tank to reduce the stress of isolation and not to the apparatus itself. To reduce the potential handling stress, 5-min of net handling was applied to fish once daily throughout the acclimatizing period. The fish were habituated to netting and transferring, in which the fish was netted and transferred to a beaker during changing of water every day throughout the acclimatization period. This was done to habituate fish to netting and

transferring as it was placed in beakers between preconditioning, conditioning, and testing. However, the condition of water, temperature, and the light was maintained the same as in the home tanks.

Pre-conditioning phase. On the day of conditioning, the fish was individually placed into the central compartment (grey) of the apparatus. After the 30s of the familiarisation period, the separators which block the yellow and white compartments respectively were removed to enable the fish to move freely for 5 min followed by 6 min of video recording to assess the basal preference of the fish by measuring the time spent in each compartment.

Conditioning. During the conditioning phase, fish were individually placed into the compartment that was initially chosen as the preferred compartment (>50% time spent) during the pre-conditioning phase, and after 5 min of settling time, AS was delivered in water followed by 5-min of video recording. Since the chemical nature of AS has not been fully characterized (4, 5), the exact concentration of AS could not be determined. However, the ratio (2 ml of AS in 5 L of water) applied consistently induced typical fear-like responses throughout the experiments. The fear parameters including erratic movement and freezing time were assessed as described above. The fish was then immediately transferred into the non-preferred compartment of the new experimental tank and 2 mL of distilled water was added to the tank. In our study, the tested fish was held in the corresponding yellow/white compartment when alarm substance/distilled water was delivered to establish an explicit relationship between the presence and absence of an aversive unconditioned stimulus and context, thus a clear safe and danger signals were presented during the trials, leading to persistence of a conditioned place aversion at 24 hours after a single CAS conditioning session.

In order to avoid the fish to be wrongly conditioned to IP and IC administration and its associated handling stress instead of AS-induced fear stimuli, fish were exposed to the conditioned stimulus (AS) for 5 mins (= fear conditioning) followed by 60 mins of the recovery period from the AS-induced stimuli. GPR139 compounds were then injected, and they were transferred to their respective home tank. Our preliminary experiments confirmed that this protocol is sufficient to archieve successful conditioning to AS-paired compartment without any interference caused by the administration of solution (control group).

Post-conditioning. On the 3rd day of the test, after the conditioning period, each fish was placed in the center compartment (grey) before the separator was removed. No AS/WD was administered during this phase. The avoidance of the AS-conditioned compartment was assessed by comparing the time spent in the AS-conditioned and unconditioned compartments for 6 min. In addition, fear-related responses of the fish in the AS-conditioned compartment were also assessed as described above.

Figures

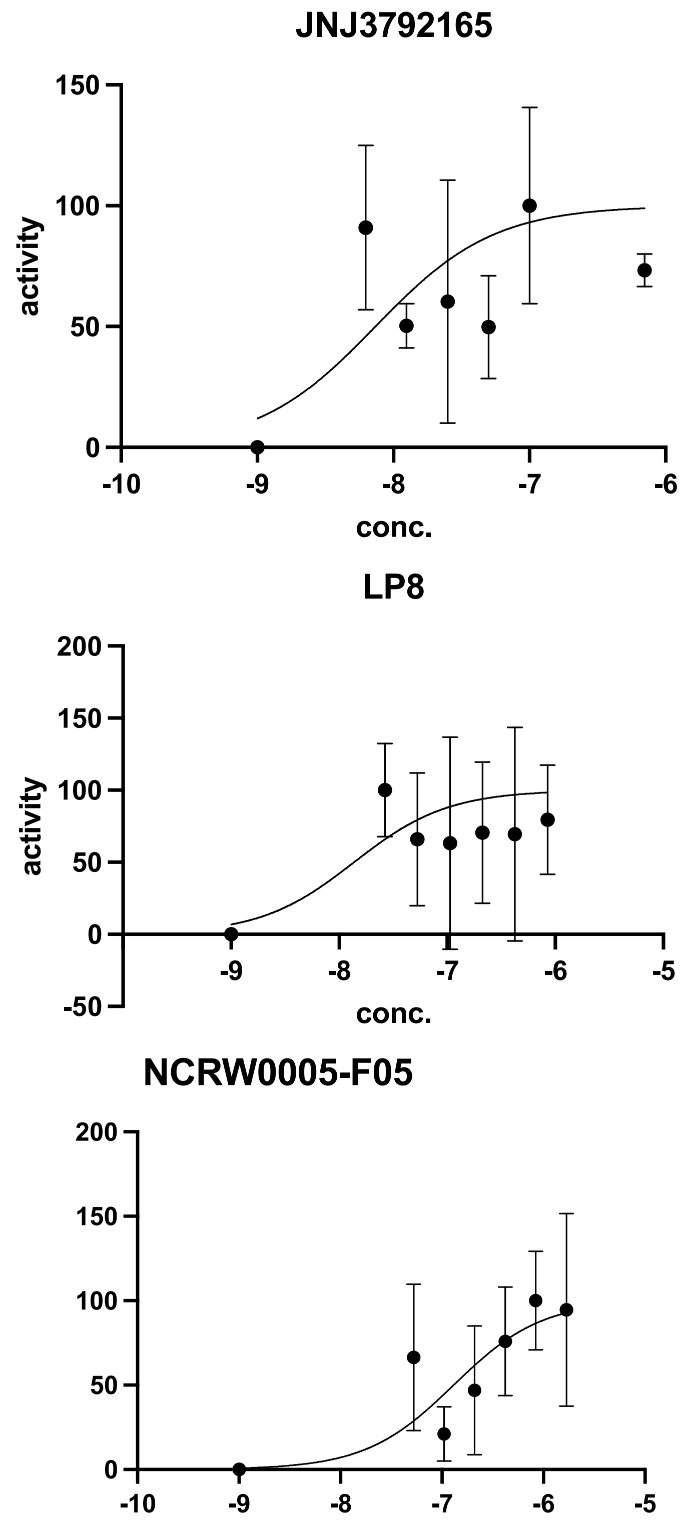

Fig S1: Dose-response curves for luciferase induction by GPR139 antagonist (JNJ3792165, LP8, NCRW0005-FO5). Graphs showing effect of GPR139 antagonists in the presence of 30 nM JNJ63533054 in HEK 293T cells expressing zebrafish GPR139. All data points are representative of three independent experiments performed in duplicate. Luciferase responses were normalized and the concentration-response curves were fitted using nonlinear regression in a sigmoidal model with variable slope according to the standard procedure provided by Graph Pad.

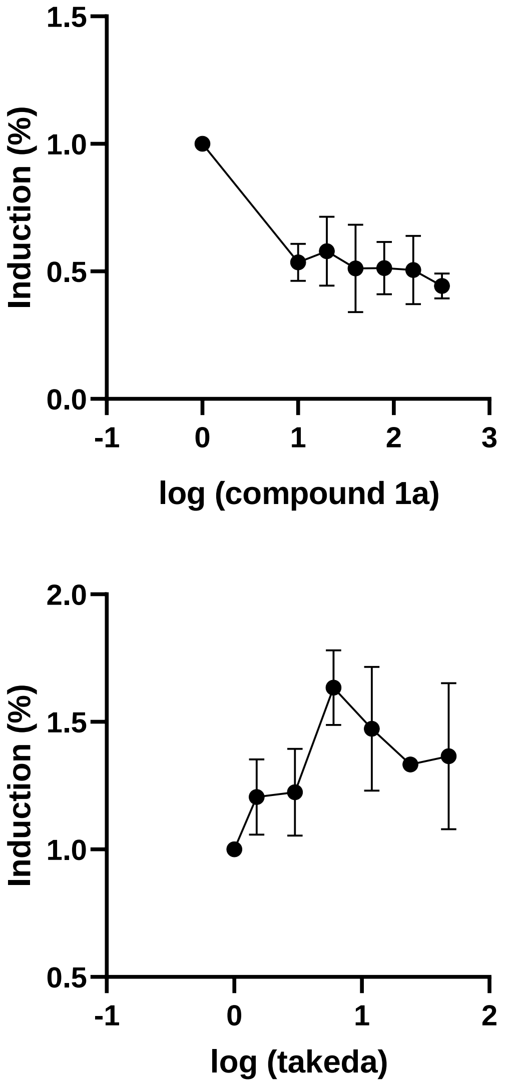

Fig S2: Dose-response curves for luciferase induction by GPR139 agonist (Compound1a, Takeda) upon zebrafish GPR139 in HEK293-T cells. Graphs showing concentration-dependent induction firefly luciferase activity divided by Renilla luciferase activity (%) in the HEK 293T cells expressing zebrafish GPR139 by Compound1a and Takeda. Luciferase responses were normalized and the concentration-response curves were fitted using nonlinear regression in a sigmoidal model with variable slope according to the standard procedure provided by Graph Pad.

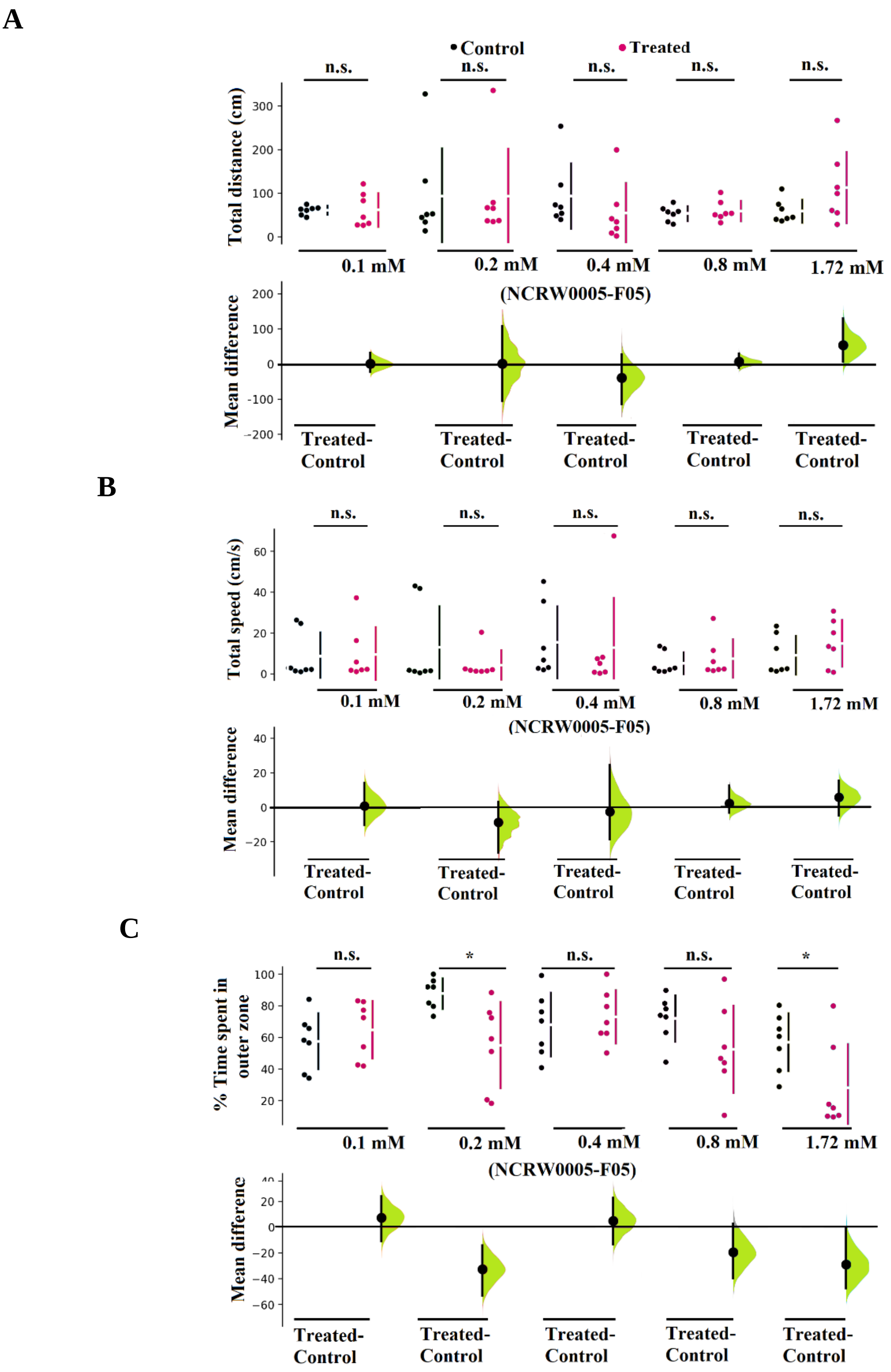

Fig S3: Toxicological effect of NCRW0005-F05 on larval zebrafish behavior. (A) There were no significant differences in total distance swam between different doses of vehicle control (DMSO) and GPR139 antagonists (0.062 % control, 0.1 mM treated, P=0.952,Cohen's *d*=0.0341, n=7; 0.125 % control, 0.2 mM, P= 0.993, Cohen's *d*=0.0066, n=7; 0.25 % control, 0.4 mM treated, P=0.324, Cohen's *d*=0.5312, n=7, 0.5 % control, 0.8 mM treated, P=0.584, Cohen's *d*=0.0939, n=7; 1 % control, 1.72 mM treated, P= 0.122 Cohen's *d*=0.8302, n=7). **(B)** There was no significant change in swimming speed (0.062 % control, 0.1 mM treated, P=0.904,Cohen's *d*=0.0682, n=7; 0.125 % control, 0.2 mM, P= 0.303, Cohen's *d*=0.6461, n=7;

0.25 % control, 0.4 mM treated, P=0.828, Cohen's *d*=0.1230, n=7, 0.5 % control, 0.8 mM treated, P=0.570, Cohen's *d*=0.1524, n=7; 1 % control, 1.72 mM treated, P= 0.323 Cohen's *d*=0.4135, n=7). **(C)** However, two of the five doses showed significant difference in total time spent in the outer zone (0.125 % control, 0.2 mM treated, P= 0.011, Cohen's *d*=1.4631, n=7; 1 % control, 1.72 mM treated, P=0.0474, Cohen's *d*=1.078, n=7). All behavioral data were analyzed using the Estimation Statistics Beta and the Statistical Package for the Social Sciences (SPSS, Version 24, IBM). All behavioral endpoints data were expressed as means±standard error of the mean (S.E.M.) and were compared by using Student's t-test, Multi two-group Cumming plot. *$p < 0.05$; **$p < 0.01$; ***$p < 0.001$; ns, not significant.

SI References

1. Maximino C, Meinerz DL, Fontana BD, Mezzomo NJ, Stefanello FV, Prestes AdS, et al. Extending the analysis of zebrafish behavioral endophenotypes for modeling psychiatric disorders: Fear conditioning to conspecific alarm response. Behavioural processes. 2018;149:35-42.
2. Ogawa S, Nathan FM, Parhar IS. Habenular kisspeptin modulates fear in the zebrafish. Proceedings of the National Academy of Sciences. 2014;111(10):3841-6.
3. Roy N, Ogawa S, Maniam R, Parhar I. Habenula GPR139 is associated with fear learning in the zebrafish. Scientific reports. 2021;11(1):1-14.
4. Parra KV, Adrian Jr JC, Gerlai R. The synthetic substance hypoxanthine 3-N-oxide elicits alarm reactions in zebrafish (Danio rerio). Behavioural brain research. 2009;205(2):336-41.
5. Mathuru AS, Kibat C, Cheong WF, Shui G, Wenk MR, Friedrich RW, et al. Chondroitin fragments are odorants that trigger fear behavior in fish. Current Biology. 2012;22(6):538-44.
